# Cholangioscopy-guided lithotripsy and extraction of gallbladder stones through the natural lumen

**DOI:** 10.1055/a-2531-9303

**Published:** 2025-02-18

**Authors:** Tong Xiao, Changqin Xu, Hongwei Xu, Yuemin Feng, Tong Su, Lechang Zhang, Shulei Zhao

**Affiliations:** 1 34708 Gastroenterology, Shandong Provincial Hospital Affiliated to Shandong First Medical University, Jinan, China

In recent years, the integration of endoscopic retrograde cholangiopancreatography (ERCP) and cholangioscopy has emerged as a prominent method for managing complex cholelithiasis 1 2 3 . We present a case demonstrating an innovative and effective approach to gallbladder stone extraction via the natural lumen ( Video 1 ).

Cholangioscopy-guided lithotripsy is performed to fragment a large stone lodged in the neck of the gallbladder, allowing subsequent complete stone clearance through the natural lumen.Video 1

A 52-year-old man presented with intermittent abdominal pain persisting over 2 months. Magnetic resonance cholangiopancreatography revealed an enlarged gallbladder with a stone lodged in its neck and additional stones in the common bile duct (CBD), a finding subsequently confirmed through direct observation with a cholangioscope ( Fig. 1 ). Given the functional status of the gallbladder and the patient's history of two prior abdominal surgeries, we opted for a natural lumen stone extraction strategy. The procedure commenced with gallbladder puncture and drainage to alleviate pressure, followed by ERCP. Two CBD stones were initially extracted using a balloon technique. Subsequently, a 9-Fr cholangioscope was navigated along the guidewire, to enable laser lithotripsy under direct visualization. Following multiple applications, the stone was successfully fragmented into smaller pieces ( Fig. 2 ).

**Fig. 1:**
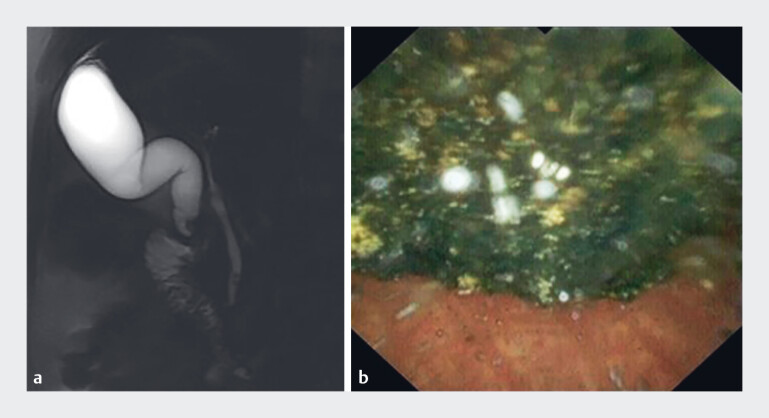
Images showing a stone of approximately 1.0 cm in size lodged in the neck of the gallbladder on: **a** magnetic resonance cholangiopancreatography; and **b** cholangioscopy.

**Fig. 2:**
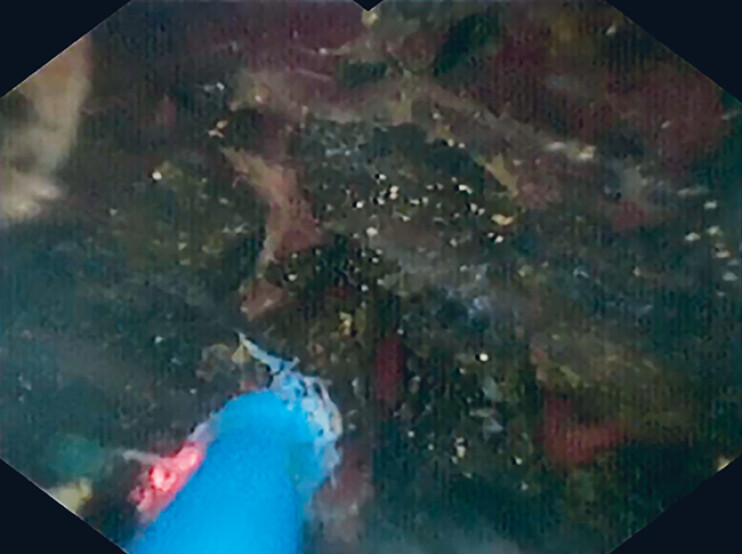
Cholangioscopic view showing the stone being fragmented into smaller pieces using laser lithotripsy.

A 10-mm × 10-cm fully coated metal stent was then positioned, with its upper part located at the gallbladder neck and the lower part exiting at the duodenal papilla. To prevent bile duct obstruction, an 8.5-Fr ×7-cm plastic stent was inserted ( Fig. 3 ). The patient experienced mild abdominal discomfort and transient amylase elevation post-ERCP, which promptly resolved with symptomatic measures.

**Fig. 3:**
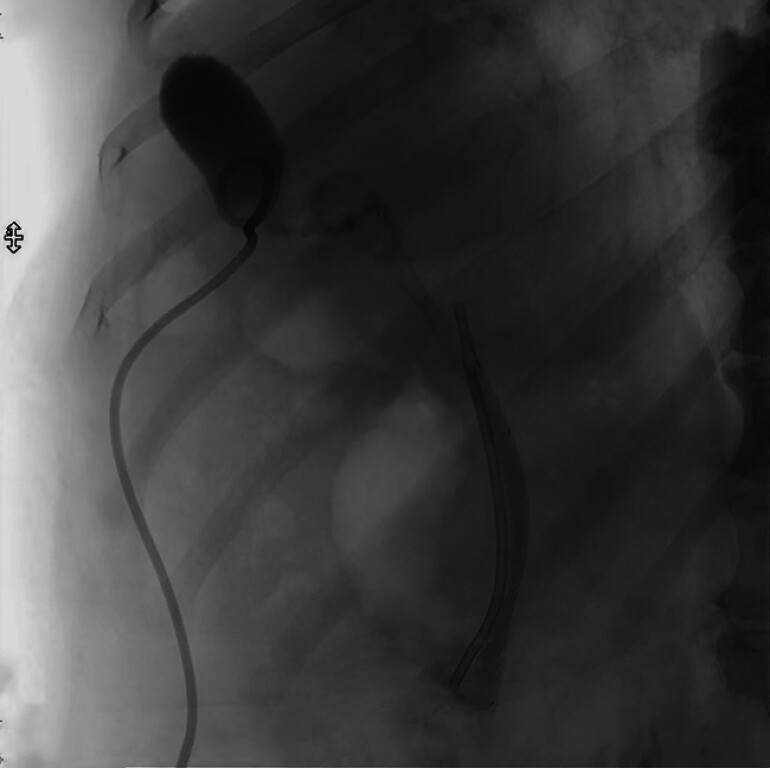
Fluoroscopic image showing the metal and plastic stents in position in the bile duct.

The gallbladder was re-accessed 4 days later using a choledochoscope passed through the metal stent. Under direct visualization, all of the remaining gallstones were extracted using a mini stone-retrieval basket ( Fig. 4 ). After complete stone clearance had been confirmed ( Fig. 5 ), both stents were removed. The patient was kept fasted for 1 day, before being discharged. A subsequent ultrasound examination 3 months later revealed no evidence of residual stones.

**Fig. 4:**
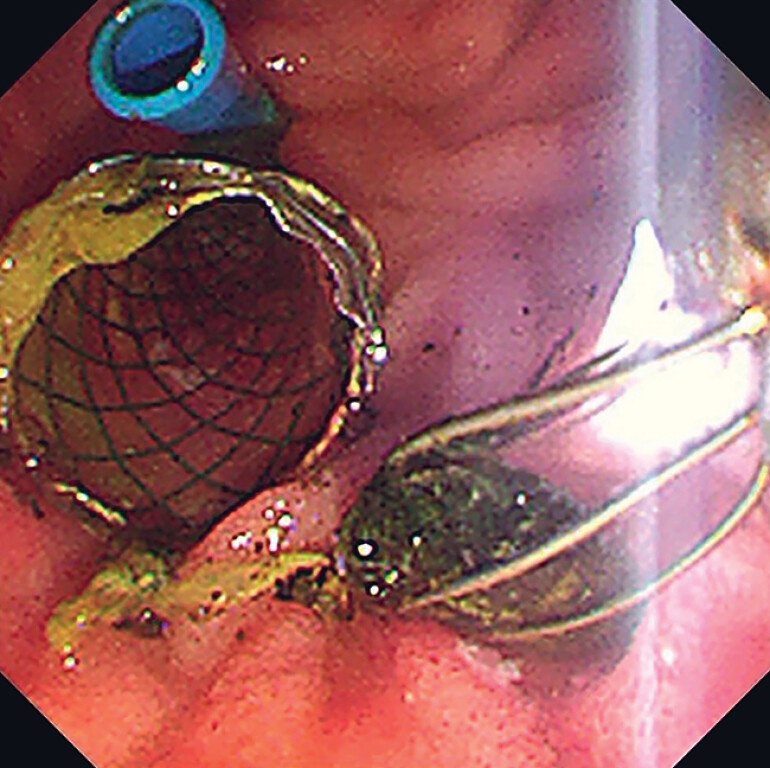
Endoscopic image 4 days after the initial procedure showing the gallstones being removed using a mini stone-retrieval basket.

**Fig. 5:**
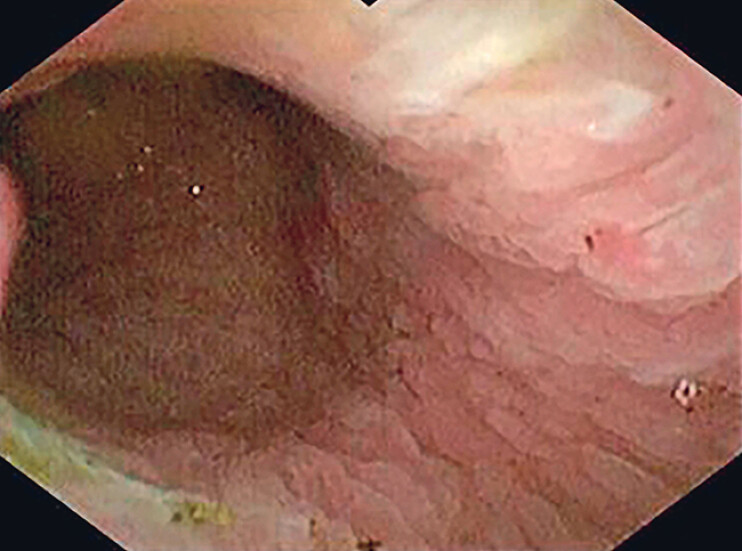
Cholangioscopic view showing no evidence of residual stones in the gallbladder.

Endoscopy_UCTN_Code_TTT_1AR_2AH

## Notes

Endoscopy E-Videos https://eref.thieme.de/e-videos
*E-Videos* is an open access online section of the journal *Endoscopy* , reporting on interesting cases and new techniques in gastroenterological endoscopy. All papers include a high-quality video and are published with a Creative Commons CC-BY license. Endoscopy E-Videos qualify for HINARI discounts and waivers and eligibility is automatically checked during the submission process. We grant 100% waivers to articles whose corresponding authors are based in Group A countries and 50% waivers to those who are based in Group B countries as classified by Research4Life (see: https://www.research4life.org/access/eligibility/ ). This section has its own submission website at https://mc.manuscriptcentral.com/e-videos .
